# RNAseq and RNA molecular barcoding reveal differential gene expression in cortical bone following hindlimb unloading in female mice

**DOI:** 10.1371/journal.pone.0250715

**Published:** 2021-10-12

**Authors:** Jordan M. Spatz, Frank C. Ko, Ugur M. Ayturk, Matthew L. Warman, Mary L. Bouxsein

**Affiliations:** 1 Center for Advanced Orthopedic Studies, Beth Israel Deaconess Medical Center, Boston, Massachusetts, United States of America; 2 University of California San Francisco School of Medicine, San Francisco, California, United States of America; 3 Harvard Medical School, Boston, Massachusetts, United States of America; 4 Boston Children’s Hospital, Boston, Massachusetts, United States of America; Kyungpook National University School of Medicine, REPUBLIC OF KOREA

## Abstract

Disuse-induced bone loss is seen following spinal cord injury, prolonged bed rest, and exposure to microgravity. We performed whole transcriptomic profiling of cortical bone using RNA sequencing (RNAseq) and RNA molecular barcoding (NanoString) on a hindlimb unloading (HLU) mouse model to identify genes whose mRNA transcript abundances change in response to disuse. Eleven-week old female C57BL/6 mice were exposed to ambulatory loading or HLU for 7 days (n = 8/group). Total RNA from marrow-flushed femoral cortical bone was analyzed on HiSeq and NanoString platforms. The expression of several previously reported genes associated with Wnt signaling and metabolism was altered by HLU. Furthermore, the increased abundance of transcripts, such as Pfkfb3 and Mss51, after HLU imply these genes also have roles in the cortical bone’s response to altered mechanical loading. Our study demonstrates that an unbiased approach to assess the whole transcriptomic profile of cortical bone can reveal previously unidentified mechanosensitive genes and may eventually lead to novel targets to prevent disuse-induced osteoporosis.

## Introduction

Mechanical loading plays an important role during musculoskeletal development and maintenance. Reduced mechanical loading from extended immobilization, spinal cord injury, or spaceflight leads to decreased bone mass and mineral density in humans, which increases susceptibility to skeletal fractures. Biological mechanisms underlying skeletal deterioration due to reduced mechanical loading have been studied using hindlimb unloading rodent models, which demonstrated altered Wnt or IGF signaling or Rank/Rankl/Opg pathway [1–5]. These studies led to several clinical studies that explored the usage of existing or newly developed pharmacological therapies to counter immobilization or spaceflight induced bone loss [6,7].

While prior studies in rodent models demonstrated signaling pathways that contribute to skeletal loss from reduced mechanical loading, limited candidate gene approached precluded exploration of other novel pathways that may also play an important role. Recent advances in whole transcriptome RNA deep sequencing (RNAseq) allow comprehensive, quantitative, and unbiased view of the complete RNA transcriptome. This high-throughput approach can allow identification novel pathways that are altered in response to reduced mechanical loading in rodent models. Prior studies in transgenic mice or mice that underwent increased mechanical loading successfully demonstrated alterations in novel pathways in osteocytes using RNAseq [8,9]. However, no studies to date examined whole transcriptome profile of osteocytes from mice that underwent reduced mechanical loading.

Thus, we examined the complete RNA transcriptome in cortical bone from mice that underwent hindlimb unloading for 7 days, with the goal of identifying novel mechanosensitive pathways that respond to mechanical unloading. We validated our RNAseq results by a highly sensitive RNA molecular barcoding technologies (NanoString) examining 60 panel genes.

## Material and methods

### Hindlimb unloading

Eleven-week old, female C57Bl/6J mice were randomly assigned to hindlimb unloading (HLU) or normal weightbearing (Cont) (n = 8/group). The HLU group underwent hindlimb unloading for 7 days according to the previously published studies [10–15]. In brief, under isoflurane anesthesia, the mouse tail was taped to a freely rotating harness connected to a wheel that moved along the central axis of the custom-made cage. The harness was adjusted such that the mouse could not touch its hind limbs to the floor or the walls of the cage. Mice were maintained on a 12/12 hour light/dark cycle, had ad libitum access to standard laboratory rodent chow and water, and were sacrificed by CO_2_ inhalation at the end of the experiment. All animal procedures were approved by and performed in accordance with the guidelines of the Beth Israel Deaconess Medical Center Institutional Animal Care and Use Committee.

### RNAseq

After cutting the ends of bones and flushing marrow with 10 ml phosphate-buffered saline, the cortical bone was placed in an Eppendorf tube and immediately snap-frozen in liquid nitrogen. Subsequently, Trizol was added while the bone is maintained frozen in an eppendorf tube placed in a liquid nitrogen bath, homogenized in the same eppendorf tube with the Fastprep24 machine (MP Biomdicals), and subsequently total RNA was isolated and purified using manufacture recommendations for the PureLink RNA kit (Life Technologies). Using this isolation technique, we routinely obtain 3–6 ug per sample total RNA that has high quality RNA integrity numbers (RIN, Aigilent Technologies) > 7.5 [9]. RNA sequencing libraries were prepared using TruSeq RNA Sample Preparation Kit (v2, Illumina, San Diego, CA) per manufacturer recommendations using 500 ng/sample total RNA [8]. Samples were multiplexed (n = 8 per lane) for sequencing on a HiSeq (Illumina) platform and reads were aligned to reference genome (Tophat2). Differential gene expression was analyzed by DESeq2 and relevant biological processes associated with differentially expressed genes were analyzed by Gene Set Enrichment Analysis according to studies by Subramanian et al. [8,16–18].

### Nanostring

To validate our set of differentially regulated genes identified by RNASeq. and to perform targeted gene discovery in our unloading model using the limiting amount of total RNA (3–6 ug/sample) we isolated from murine femurs, we used a NanoString nCounter codeset of sixty differentially expressed genes (Table 1) with 6 housekeeping genes (Actb, Abcf1, B2m, Gapdh, Pol42A, Sirt4). In brief, the nCounter gene codeset contains a matched pair 3′ biotinylated capture probe and a 5′reporter probe tagged with a fluorescent barcode, for each of 236 transcripts. Probes are hybridized to 100 ng of total RNA for 19 h at 65°C, after which excess capture and reporter probes are removed and transcript-specific ternary complexes are immobilized on a streptavidin-coated cartridge. Subsequently, all solution manipulations are carried out using the NanoString preparation station robotic fluids handling platform. Data collection is carried out with the nCounter Digital Analyzer to count individual fluorescent barcodes and quantify target RNA molecules present in each sample. Normalization was carried based on a standard curve constructed using spike in exogenous control constructs and the 6 housekeeping genes included in the codeset [19].

**Table 1:** Differentially expressed genes in the control and hindlimb unloaded (HLU) mouse cortical bone assessed by NanoString.

Molecular Pathways	Selected Genes	Differentially Expressed Genes	HLU vs. Control (Fold Change)	p-value
Mesenchymal stem cell (MSC) fate determination and differentiation	Abi3bp, Fabp4, Apod			
Osteoblast function and differentiation	Aspn, Alpl, Bmpr1a, Dkk1, Fzd4, Tgfb1-3, Runx2, Tob1, Opg, Spp1, Sp7, Sparc, Bglap, Myoc, Col1a1, Col1a2, Col3a1, Den, Serpinfl, Sfrp2, Sfrp4, Wnt16, Wnt9a, Wnt5a, Wnt4, Wisp2, Zfyve9, Snca, Bmp4	Fzd4 Sfrp2 Sfrp4 Spp1 Bmp4	1.6 1.7 1.7 1.5 1.4	0.03 0.05 0.009 0.02 0.02
Osteocyte function and differentiation	Sost, Mef2C, Mepe, Postn, Phex, Npy	Npy	1.2	0.04
Osteoclast function and differentiation	Ctsk, Tnfsfl1(Rankl), Trap (Acp5)			
Extracellular matrix proteases	Mmp2, Mmp3, Mmp10, Mmp13, Mmp14, Mmp15	Mmp3 Mmp13	1.8 1.6	0.007 0.01
Protease inhibitors	Timp1, Timp2	Timp1	1.5	0.02
Cell cycle control, mitochondria, energy balance	Mss51, Scd1, Pfkfb3	Scd1 Pfkfb3	2.0 1.8	0.01 0.004
Housekeeping genes	Actb, Abcf1, B2m, Gapdh, Pol42A, Sirt4			

## Results

**Fig 1:**
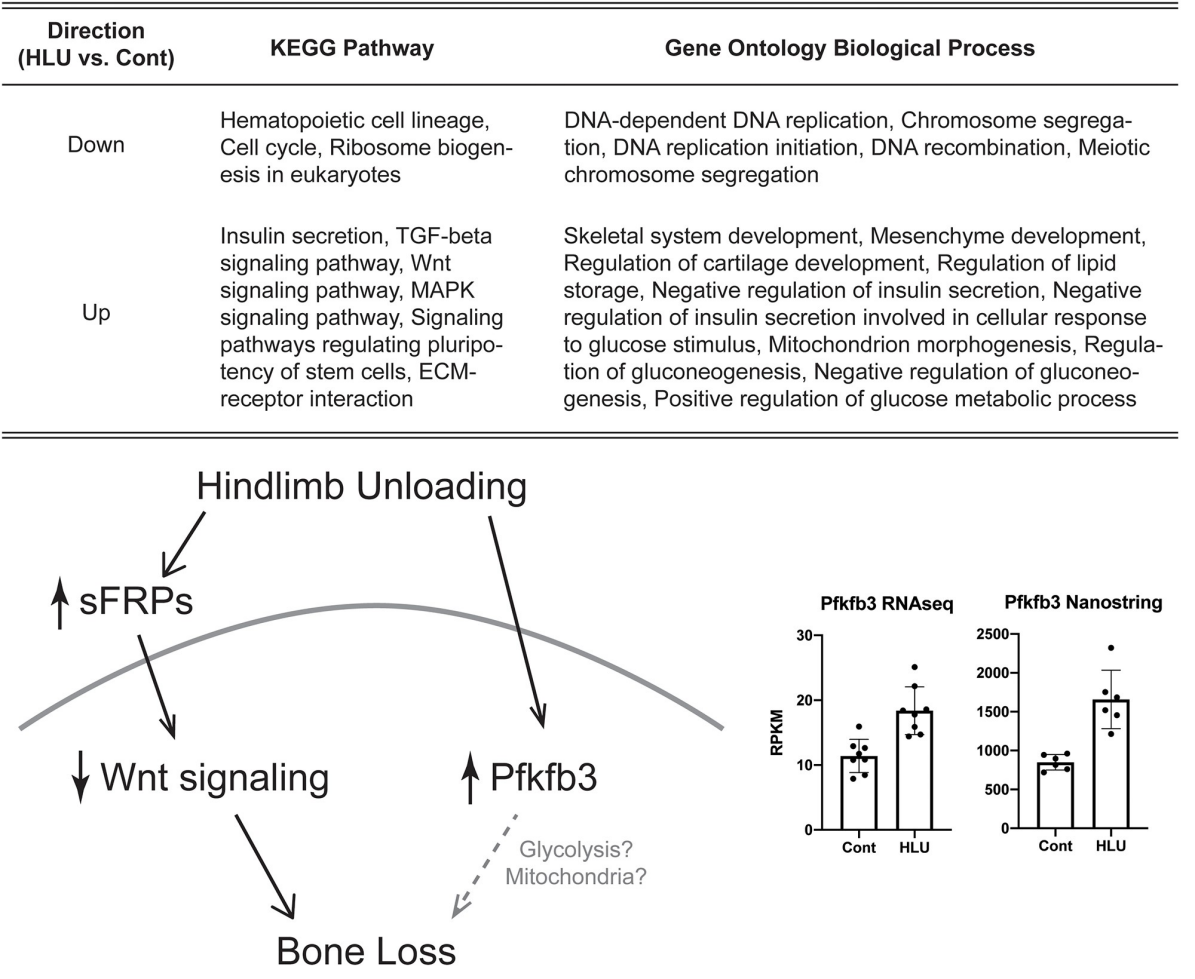
Selected KEGG pathway and gene ontology of biological process altered in cortical bone from hindlimb unloaded mice and the effects of HLU on Wnt signaling and Pfkfb3.

Hindlimb unloading did not alter the quality of RNAseq data. For both groups, individual specimens yielded ~22 million reads with high unique mapping rates (79% for controls and 81% for HLU). RNAseq analysis of cortical bone mRNA from control and HLU mice, without correcting for multiple hypothesis testing, identified 723 genes whose transcript abundances increased ≥ 1.2-fold and 610 genes whose transcript abundances decreased ≥ 1.2-fold. After considering genes whose transcript abundances changed by 1.2-fold and correcting for multiple hypothesis testing (p < 0.1), 8 genes demonstrated increased transcript abundance and 5 genes demonstrated decreased transcript abundance following HLU. Gene set enrichment analysis using these 13 differentially expressed genes pointed to several metabolic processes, monosaccharide metabolism (Pfkfb3, Igfbp5, p < 0.01), peptidase inhibition (Stfa1, Stfa2, p < 0.01), and cellular protein metabolism (Stfa2, Stfa1, Igfbp5, p < 0.01). Wnt signaling was also implicated (Fig 1).

**Fig 2:**
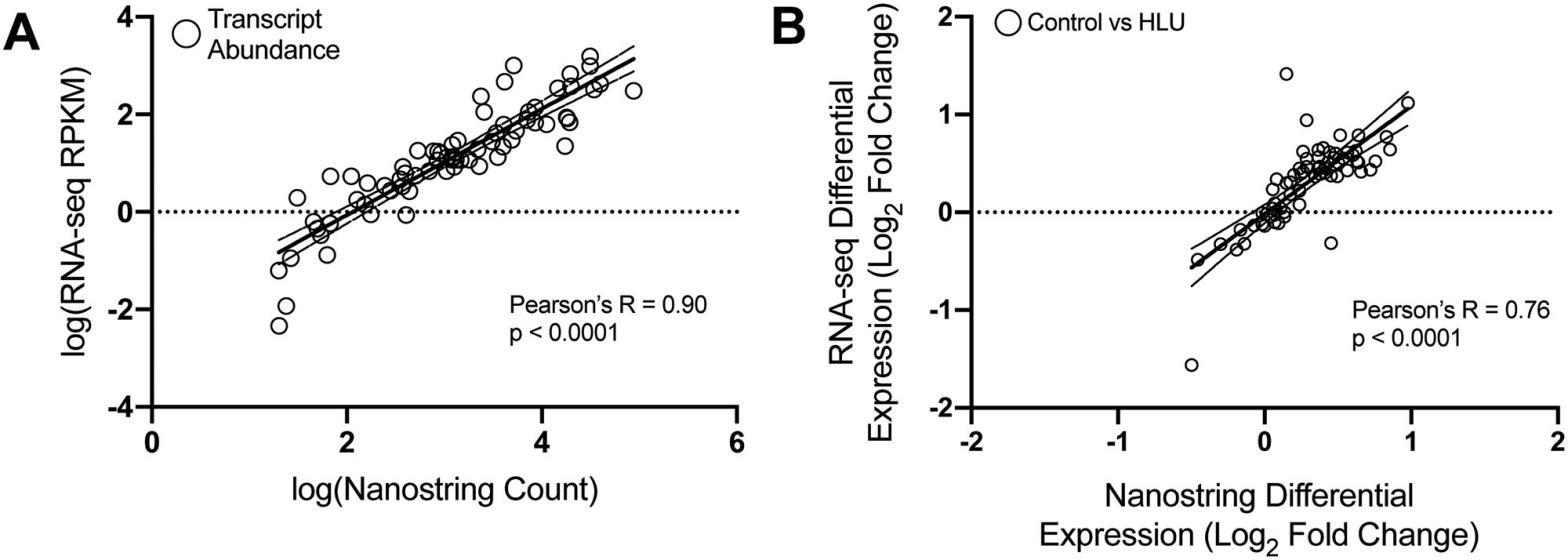
Correlation between Nanostring and RNAseq transcript abundance in controls (A) and changes in abundance between control and HLU mice (B) with 95% confidence bands of the best-fit line.

**Fig 3:**
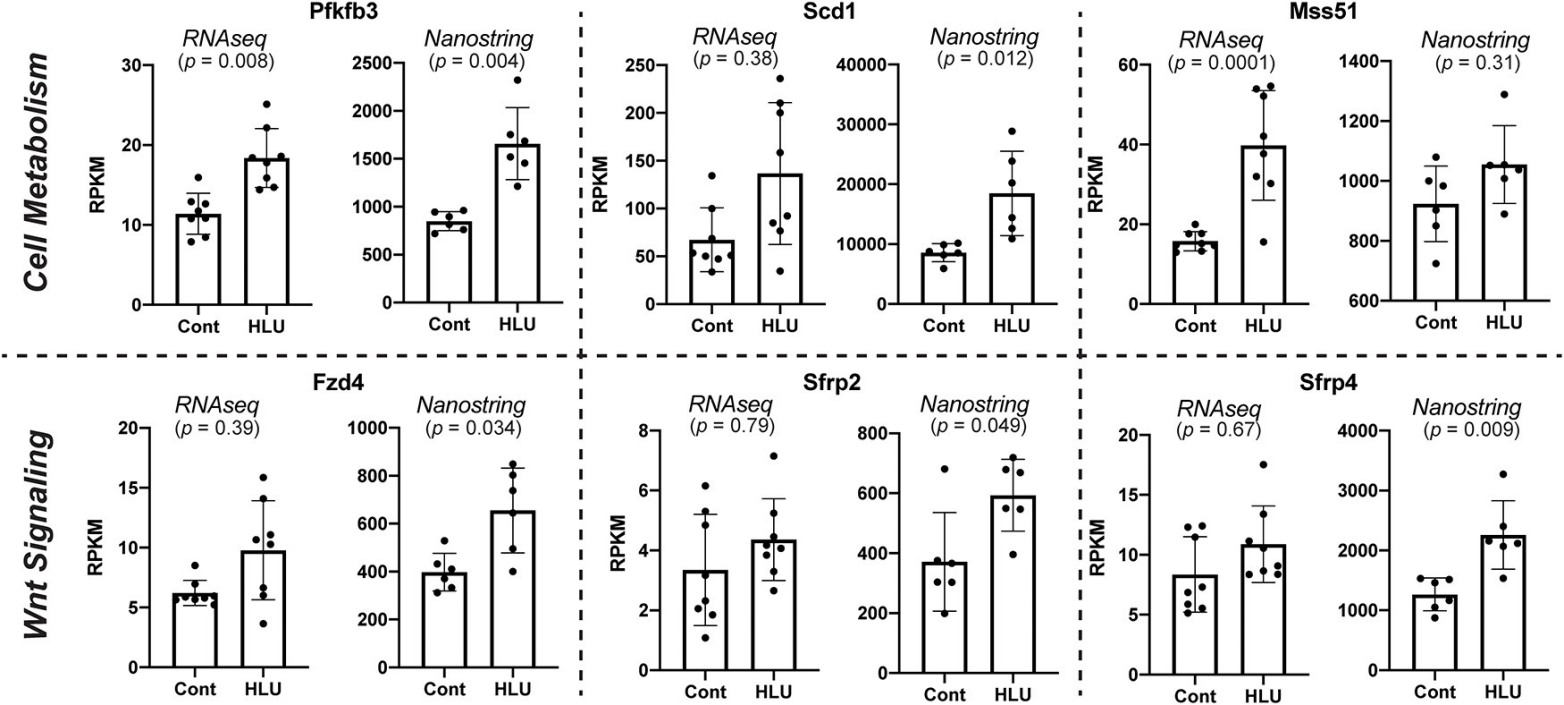
Cell metabolism and Wnt signaling associated transcripts whose abundance changed between control and HLU mice, as assessed by RNAseq and Nanostring analysis.

Transcript abundances measured by RNAseq and Nanostring were similar in control mice (Pearson’s R = 0.90, p < 0.0001, Fig 2A). Changes in transcript abundance between control and HLU mice also correlated significantly between the RNAseq and Nanostring datasets (Pearson’s R = 0.76, p < 0.0001, Fig 2B). Importantly, Nanostring confirmed alterations seen with RNAseq in transcripts associated with Wnt signaling and cell metabolism (Table 1, Fig 3). Scd1 (2-fold, p = 0.01), Pfkfb3 (1.8-fold, p = 0.003), and Fzd4 (1.6-fold, p = 0.03) were all upregulated in cortical bone isolated from hindlimb unloaded mouse. In addition to these genes, we identified Mmp3 (1.8-fold, p = 0.007), Sfrp2 (1.7-fold, p = 0.05), Sfrp4 (1.7-fold, p = 0.009), Mmp13 (1.6-fold, p = 0.01), Bmp4 (1.4-fold, p = 0.02), Timp1 (1.5-fold, p = 0.02), Npy (1.2-fold, p = 0.04), and Spp1 (1.5-fold, p = 0.02) genes to be upregulated in the hindlimb unloaded mouse cortical bone. No downregulated genes were detected.

## Discussion

Our RNAseq dataset provides an unbiased approach for identifying genes whose transcripts change in response to mechanical unloading of cortical bone. We identified novel genes (e.g., Mss51, Pfkfb3) related to cell cycle, mitochondria, and cellular energy balance that were differentially regulated by HLU.

While mitochondria are typically reduced in size as osteoblasts transition to osteocytes, histopathological studies demonstrate enlarged mitochondria in osteocytes from rats immobilized for ten days [20]. Further, in mice with impaired osteocyte autophagy, enlarged mitochondria are observed as well, and associated with decreased trabecular and cortical bone [21]. Imbalance in osteocyte mitochondrial redox can disrupt the canalicular network and lead to bone loss [22]. Further studies are needed to determine whether the genes we identified may sense altered mechanical loading through a similar mechanism that regulates cellular energy balance in bone.

Prior studies have also demonstrated that microgravity causes alterations to glycolysis pathways in a variety of cells, including osteoblasts [23,24]. Importantly, our data show that Pfkfb3, which controls the concentration of fructose 2,6-bisphosphate, a potent allosteric activator of PFK1, is significantly upregulated after HLU. Recent evidence supports the concept that Pfkfb3 provides a signaling mechanism for Wnt3A, altering osteoblast differentiation [25]. Pfkfb3 was also identified de-novo in a gene enrichment analysis of circulating monocytes in patients with osteoporosis, further suggesting a possible role in modulating the bone microenvironment in the setting of low bone density [26]. Our study adds to the evidence that Pfkfb3 may influence bone metabolism in the context of microgravity, disuse-induced bone loss and osteoporosis.

Mss51 has not been demonstrated to be involved during skeletal development or homeostasis, but recently published study suggests that Mss51 may be involved in bone adaptation following mechanical loading [27]. The alteration in Mss51 appears to be mediated by focal adhesion kinase, which has been shown to be responsive to mechanical loading via fluid flow shear stress in osteoblasts [28,29]. Mechanical loading likely alters cell-matrix environment, thereby allowing integrins to initiate intracellular signaling to promote bone adaptation. Hindlimb unloading in rats also decreases focal adhesions in mesenchymal stromal cells [30], which will have resulted in increased Mss51 [27]. These studies demonstrate that while Mss51 is conventionally known to be involved during cell metabolism and energy balance, the gene may also be responsive to mechanical loading/unloading via changes in cell-matrix environment.

Prior studies of hindlimb unloading in rodents have reported that several signaling pathways such as Wnt and Rankl/Opg are altered in osteoblasts and osteocytes following mechanical unloading [31–34]. While our study demonstrated that Wnt signaling is implicated to be involved, we did not find that sclerostin or Rankl/Opg to be altered following hindlimb unloading. There are several possible factors that may have contributed to differences in study findings. First, it is possible that methodological differences (RNAseq vs. RT-qPCR) used to assess transcript levels contributed to different outcomes. Second, while we carefully removed bone marrow, surrounding soft tissues, and both ends of bone, the cortical bone lining cells may have contributed to heterogeneity of cell population, leading to blunted effects on several signaling pathways known to be altered by hindlimb unloading in osteocytes or osteoblasts. In addition, whereas we examined day 7 of unloading, earlier time points may be needed to observe robust differences in gene expression due to unloading. Prior studies have demonstrated that bones from 6 to 9 months old rats are more responsive to hindlimb unloading than 28 to 32 months old rats, likely due to increased Bmp2 and Igf1 in younger animals [35,36]. Finally, hindlimb unloading has been shown to affect other physiologic systems in female 11-week-old mice, such as loss of muscle and increased corticosterone metabolites [37], which has been shown to inhibit bone formation [38]. Altogether, a variety of factors can contribute to inconsistencies in gene expression across studies. Additional suitably powered and well-controlled studies are needed to resolve reported discrepancies among studies.

We used only female mice, thus precluding analysis of possible sexual dimorphism in mechanosensitive genes. We selected the 7-day timepoint based on our prior studies, but recognize the limitation of this approach which precludes determining the time-course of gene-expression changes. This early timepoint may have led to minimal changes in osteogenic genes in our studies. However, our ability to identify novel genes that may initiate osteogenic responses later, not previously associated with disuse-induced bone loss provides rationale for future studies with multiple timepoints to expand upon the current findings. While there was excellent correlation between the measurements performed with two methods (Fig 2), Nanostring detected more differentially expressed genes than RNAseq (Fig 3). Therefore, although Nanostring can sample a limited, pre-determined portion of cortical bone transcriptome, it can serve as a powerful approach towards identifying mild changes (<2-fold) in transcript abundance.

Our gene expression approach comparing cortical bone gene expression in HLU and normally loaded mice successfully revealed previously known and unknown genes. Future mechanistic studies examining the functional role of metabolic genes revealed from our study may identify novel mechanosensitive pathways responsible for microgravity- and disuse-induced bone loss. Elucidating these pathways may lead to therapeutic interventions to treat bone loss and prevent fracture risk associated with microgravity, spinal cord injury, or extended bed rest.

## Supporting information

S1 File Nanostring supple. (XLSX)

S2 File RNAseq supple. (CSV)
